# Using Google AdWords for International Multilingual Recruitment to Health Research Websites

**DOI:** 10.2196/jmir.2986

**Published:** 2014-01-20

**Authors:** Margaret S Gross, Nancy H Liu, Omar Contreras, Ricardo F Muñoz, Yan Leykin

**Affiliations:** ^1^University of CaliforniaSan Francisco, CAUnited States; ^2^Palo Alto UniversityPalo Alto, CAUnited States

**Keywords:** Internet recruitment, multinational, online, Internet research, recruitment costs

## Abstract

**Background:**

Google AdWords, the placement of sponsored links in Google search results, is a potent method of recruitment to Internet-based health studies and interventions. However, the performance of Google AdWords varies considerably depending on the language and the location of the target audience.

**Objective:**

Our goal was to describe differences in AdWords performance when recruiting participants to the same study conducted in four languages and to determine whether AdWords campaigns can be optimized in order to increase recruitment while decreasing costs.

**Methods:**

Google AdWords were used to recruit participants to the Mood Screener, a multilingual online depression screening tool available in English, Russian, Spanish, and Chinese. Two distinct recruitment periods are described: (1) “Unmanaged”, a 6-month period in which ads were allowed to run using only the AdWords tool itself, with no human intervention, and (2) “Managed”, a separate 7-week period during which we systematically sought to optimize our recruitment campaigns.

**Results:**

During 6 months of unmanaged recruitment, our ads were shown over 1.3 million times, resulting in over 60,000 site visits. The average click-through rate (ratio of ads clicked to ads displayed) varied from 1.86% for Chinese ads to 8.48% for Russian ads, as did the average cost-per-click (from US $0.20 for Chinese ads to US $0.50 for English ads). Although Chinese speakers’ click-through rate was lowest, their rate of consenting to participate was the highest, at 3.62%, with English speakers exhibiting the lowest consent rate (0.97%). The conversion cost (cost to recruit a consenting participant) varied from US $10.80 for Russian speakers to US $51.88 for English speakers. During the 7 weeks of “managed” recruitment, we attempted to improve AdWords’ performance in regards to the consent rate and cost by systematically deleting underperforming ads and adjusting keywords. We were able to increase the number of people who consent after coming to the site by 91.8% while also decreasing per-consent cost by 23.3%.

**Conclusions:**

Our results illustrate the need to linguistically and culturally adapt Google AdWords campaigns and to manage them carefully to ensure the most cost-effective results.

## Introduction

The rapid advance of digital media is changing the way individuals interact with and consume information. Digital media is becoming ubiquitous in many parts of the world, quickly outpacing traditional media (eg, newspapers) [1,2]. Given the rapid growth and increasing popularity of digital media, it is important for researchers interested in recruiting representative samples to remain informed about the efficient and effective ways of utilizing the instruments afforded by digital media for recruitment into research studies [3].

One of the chief advantages of digital media is that much of it is available for free; the costs of media production are offset by revenue that comes largely from advertisement. A company that might be considered a preeminent example of such model is Google, which provides a variety of digital media services for consumers free of charge by having a powerful and successful platform to sell advertisements that are displayed to consumers of these services. Indeed, in the past few years, Google has been consistently ranked as the world’s most popular search engine [4]. Google, with over 660 million daily visitors is also one of the world’s most popular websites in general [5], used for information gathering, shopping, email, navigation, cloud file storage, social networking, translation, and more. This robust “ecosystem” combined with Google’s immense popularity appeals to advertisers, who use Google AdWords—Google’s advertising service—to create and carry out their ad campaigns. Google’s popularity can also benefit researchers aiming to recruit participants into their studies quickly and inexpensively [6]. With such a large proportion of Internet users relying on Google services, it is possible that using Google AdWords for recruitment might yield samples that are reasonably representative of Internet users [7,8].

**Figure 1:**
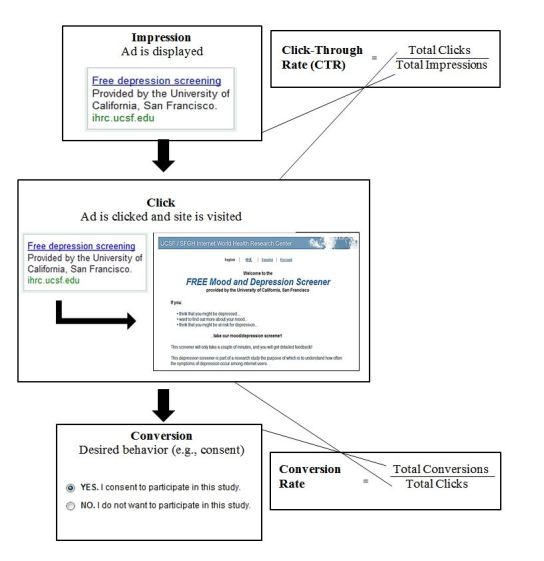
Google AdWords terms.

Though created for advertising products and services, Google AdWords has a robust set of tools that can facilitate recruitment into research. These include customizations related to keywords that trigger the ads, targeting specific geographical areas, timing of ads, and the ability to conduct multiple campaigns with distinct options concurrently. Thus, researchers may be able to recruit only in neighborhoods of interest, and only among individuals already looking for specific information (eg, depression treatment). To facilitate the evaluation of performance of campaigns (eg, to determine whether a specific recruitment message appeals to the audience of interest), AdWords has a range of tools that evaluate specific ads and specific keywords. These include calculations of *impressions* (instances when an ad is displayed to the user), *clicks* (instances when user clicks on an ad), and *conversions* (instances when a desired behavior occurs, eg, a purchase, or, in case of research, a consent). AdWords automatically calculates the *click-through rate* (CTR: ratio of total clicks per total impressions), and *conversion rate* (CR: ratio of total conversions per total clicks). These terms and concepts are illustrated in Figure 1.

AdWords also has tools to monitor and manage the cost of ad campaigns. Costs are incurred whenever a displayed ad is clicked. Displaying the ad (an impression) does not cost money, however, the position of the ad (and therefore its visibility for users) is determined by the maximum amount of money an advertiser is willing to pay for the ad to be clicked, in an auction bidding system. Thus, if two ads from two advertisers could be displayed for a given keyword, the ad for which an advertiser is willing to pay more will be displayed first, increasing the likelihood of it being clicked (the actual cost of the click is US $0.01 greater than the next highest bid). For popular keywords like “depression”, research studies need to compete with advertisers who are willing to expend considerable funds for clicks (eg, pharmaceutical companies). Thus, to make the most of the limited budget of research studies as well as other ad campaigns, AdWords can suggest keywords that are likely to generate interest (ie, clicks and conversions) and will automatically preferentially display the ads to improve click counts or CRs (depending on the goal of the campaigns), based on data from past ad performance and Google’s own algorithms. However, AdWords recruitment has several drawbacks—chief among which are the strict limit on the number of characters and the fact that costs are calculated based on clicks (visits) rather than actions (eg, consent). Taken together, these drawbacks can add up to a costly campaign for studies that are difficult to describe concisely, where visitors may feel misled by the advertisement.

This paper describes our experience conducting a multilingual worldwide campaign to recruit participants to a website that screens for symptoms of depression, in four languages. We will present the results from two distinct time periods of recruitment. First, a 6-month “Unmanaged” recruitment period, in which AdWords campaigns were allowed to run only with automated optimizations offered by the AdWords tool, with no human intervention. For this period, we will describe similarities and differences in campaign performance between the four languages of recruitment (English, Spanish, Russian, and Chinese). During the second “Managed” period (a separate 7-week period), we systematically sought to optimize our recruitment campaigns beyond the optimization provided by automated AdWords tools, with the goal of increasing our conversion rate (ie, consent rate) while decreasing our costs.

## Methods

### Participants

Eligible participants were 18 years of age; no other condition was necessary for eligibility. Participants were speakers of either English, Spanish, Chinese, or Russian. Two distinct waves of recruitment are described: a 6-month “Unmanaged” period and a 7-week “Managed” period. During the “Unmanaged” recruitment period (no management of ads), over 60,000 people visited the Mood Screener website, 30,218 were screened for eligibility, and 26,194 individuals were eligible to participate. Among eligible participants, 3828 were English speakers, 7477 were Spanish speakers, 5395 were Chinese speakers, and 9494 were Russian speakers. Eligible participants were on average 30.9 years of age (SD 11.2), 68% (17,811) were women, and 57% (14,930) identified as white or Caucasian.

During the “Managed” recruitment period (active management of ads), over 19,000 people visited the Mood Screener website, 7786 were screened for eligibility, and 6564 individuals were eligible to participate. Among eligible participants, 1282 were English speakers, 2350 were Spanish speakers, 589 were Chinese speakers, and 2343 were Russian speakers. Eligible participants were on average 31.3 years of age (SD 12.0), 66% (4332) were women, and 47% (3085) identified as white or Caucasian.

### Materials

The Demographics - 1 questionnaire asked about participants’ age, gender, race, and country of residence. The Demographics - 2 questionnaire asked about participants’ country of birth (if different from country of residence), whether they lived alone or with others, as well as their education, employment status, marital status, income, subjective social status (perceived position in society, ie, the “social ladder” [7]), and history of seeking treatment for depression.

The MDE Screener [9] is an 18-item self-report measure based on Diagnostic Interview Schedule items [10] designed to screen for the presence of current and past major depressive episodes (MDEs). It rates the presence of nine symptoms of depression according to the DSM-IV [11] over a period of 2 weeks or more and assesses whether Criterion C (significant impairment in functioning) is met within the same time span. Participants are asked about the presence of symptoms over the past 2 weeks (to identify a “Current” MDE), as well as symptoms occurring in any 2-week period in their lifetime, excluding the past 2 weeks (to identify “Lifetime” MDE). The screener has been shown to have good agreement with the PRIME-MD [12,13] and with clinician-administered diagnostic interviews [14].

### Overall Study Procedures

Study procedures have been described in detail elsewhere [15]. Briefly, worldwide Google AdWords campaigns were used as the main recruitment source. Recruitment was made possible by a Google grant awarded to our research group, which permitted us to run recruitment campaigns for our investigations, with a maximum bid of US $1 per click. This US $1 limit made our ads less competitive in markets with significant AdWords saturation (eg, Western English-speaking world), but reasonably competitive in other parts of the world.

Our ads were triggered by individuals entering terms related to depression or sad mood into the Google search engine; the ads were keyed to the language of the keyword. Google AdWords limits their ads to 25 characters for the title line and 35 characters for each of two body lines (for Chinese-language ads, the limits are 12 and 17 characters for title and body, respectively, as Chinese characters are considered “double width”). Clicking on the ad forwards the user to the first (“landing”) page of the site in the appropriate language; this page contained information on the nature of the site as well as limits to confidentiality. Those interested in continuing advanced to the Demographics – 1 questionnaire (age, race, gender, and country of residence). Eligible participants (those 18+ years of age) then advanced to the MDE Screener, which was followed by the “honesty question” that asked whether participants’ responses are “accurate” or whether participants were “testing the site”. Participants completing “Current” MDE Screener with no more than 2 missing answers were given personalized feedback (brief explanation of the symptom level as well as additional feedback for those indicating suicidality). Participants were then invited to take part in a monthly rescreening study. Interested individuals read the consent document and signed it with their email address. They then completed the Demographics – 2 questionnaire and the “Lifetime” MDE screener, followed by personalized feedback. Starting in February 2013, consenting participants indicating current or past suicidality also completed a detailed suicidal behaviors assessment. Consenting participants were then contacted via email monthly with a link to return to the site to rescreen their mood.

### AdWords Management Procedures

This investigation focuses on two distinct recruitment periods. The first, “Unmanaged”, was the 6-month period from November 15, 2011, to May 15, 2012, when no active ad management was taking place, although automatic Google-based ad management was still in effect (via enabling the setting for showing ads that are expected to maximize conversions). The study was then paused while we transitioned to a different software vendor and was restarted in February 2013. We restarted the ads that were in place during our previous recruitment period and started managing them actively shortly thereafter. Thus, the second period, “Managed”, was the 7 weeks from March 21 to May 8, 2013, when we managed ads actively, focusing on increasing conversion (consent) rates. In order to do this, we systematically examined the performance of each ad and disabled the ones that (1) had produced no or very few conversions, because these ads were unlikely to produce future conversions or (2) had high CTRs, but low CRs, because these ads appeared to be attracting those unlikely to consent.

In both cases, our goal was to minimize the opportunity cost of presenting ads that were unlikely to result in a conversion and retain the ads that were most productive in terms of conversions. For each language, we monitored the campaign weekly performance and suspended (paused) the 2-4 worst performing ads (according to the above criteria) each week.

To further optimize performance, we also managed the keywords during this 7-week period. Similarly to ad management, we focused on the performance of each individual keyword and disabled the ones that produced no or very few conversions or had high click counts, but low CRs. In addition to deleting poorly performing keywords, we continuously added keywords including misspelled variations of successful keywords (eg, “depressd” and “depresed”). This was useful to our campaign due to the high frequency of searches containing misspelled words or phrases combined with much less competition among other Google AdWords campaigns.

This study was approved by the University of California, San Francisco Institutional Review Board (Committee for Human Research).

## Results

### Overview

For the purpose of this study, ads that had the highest numbers of conversions were considered to be the best performing ads. Conversely, if an ad was being clicked but was not generating conversions, the ad was considered low-performing.

### Unmanaged Period

The summary of the ad campaign statistics are presented in Table 1. Chinese ads had by far the highest total impressions (602,634, with other languages ranging from 241,507 to 301,493), which is perhaps indicative of both the number of Chinese speakers on the Internet and the demand for information on depression among the Chinese-speaking Internet community. Despite the high number of impressions, Chinese ads generated relatively few clicks (11,209), resulting in the lowest CTR (1.86%). However, the consent rate for Chinese speakers was the highest (3.62%, with other languages ranging from 0.97% to 2.46%), resulting in the second highest number of consenting participants (406).

Although the Russian campaign had the second fewest impressions (227,391), it had by far the highest number of clicks (23,514, with other languages ranging from 11,022 to 14,459), resulting in the highest CTR of all languages (8.48%, with other languages ranging from 1.86% to 5.99%). Russian cost-per-click was likewise the lowest (US $0.20, with other languages ranging from US $0.33 to US $0.50), suggesting very low competition for ads for this language. Surprisingly, although this campaign had the highest CTR, it also had the second lowest consent rate (1.87%), which nonetheless (due to the high number of initial clicks) produced 440 consented participants.

The English campaign was mostly notable for its highest costs, both per click (US $0.50) and especially per consent (US $51.88), which was almost 5 times the costs for the other languages, which ranged from US $10.80 to US $13.40. The rate of consent among English speakers was the lowest (0.97%), resulting in a far lower number of consenting participants than other languages (107, whereas other languages ranged from 355 to 440).

The Spanish campaign resulted in 355 consented participants with the second highest consent rate (2.46%). This campaign had 241,507 impressions, 14,459 clicks*,* and the second highest CTRs (5.99%). The cost-per-consent for the Spanish ads was US $13.40.

We also noted both similarities and differences in the keywords (ie, search terms that trigger ads) that were the most effective in generating the most conversions (see Table 2). The most successful keyword in Russian, Chinese, and Spanish was “depression”, while the English campaign’s use of “depression” produced very few conversions; “depression test” was the most effective keyword. This is likely due to the prevalence of competing English ad campaigns that also use the keyword “depression”. Indeed, “depression test” or its variations (eg, “test de depresión”) was one of the 4 most productive keywords in all languages except Chinese. Chinese campaigns were unique in that the characters for “suicide” and “commit suicide” (Table 2) were 2 of the 4 most productive keywords, whereas no other language had “suicide” as one of the top 4 keywords (Russian keyword “не хочеться жить”, “do not want to live”, is perhaps an exception). Finally, Spanish was the only language where “anxiety” (“la ansiedad”) appeared as a top keyword, and Russian was the only language where non–mood-related keyword “психологические тесты” (“psychological tests”) was a top keyword. A list of worst performing keywords is included in Table 3.

Finally, for each language there was a particular ad that significantly outperformed other ads with respect to conversions (see Table 4). The English, Russian, and Spanish campaigns’ most successful ads all contained the word “free” when describing the study. All four languages’ most successful ads contained the name “University of California, San Francisco”, or its abbreviation, and were direct in describing the purpose of the study. Ads that contained less formal descriptions (eg, “Can’t eat, sleep, focus?”) were less successful (Table 5).

**Table 1:** Statistics from the unmanaged 6-month period.

	English	Russian	Chinese	Spanish	Overall
Impressions, n	301,493	277,391	602,634	241,507	1,373,025
Clicks (visitors), n	11,022	23,514	11,209	14,459	69,204
Click-through rate, %	3.66	8.48	1.86	5.99	4.38
Cost-per-click, US$	0.50	0.20	0.42	0.33	0.33
Consented, n	107	440	406	355	1,308
Consent rate, %	0.97	1.87	3.62	2.46	2.17
Cost per consent, US$	51.88	10.80	11.71	13.40	15.15

**Table 2:** Best performing keywords.

Language	Best performing keywords	English translation
English	depression test	
	sad	
	symptoms of depression	
	Am I depressed	
Russian	депрессия	depression
	психологические тесты	psychological tests
	депрессия тест	depression test
	не хочеться жить	do not want to live
Chinese	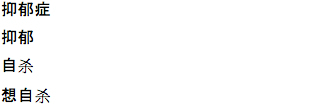	depression
		depression
		suicide
		wanted to commit suicide
Spanish	Depresión	depression
	Test de depresión	depression test
	La ansiedad	anxiety
	Síntomas de depresión	symptoms of depression

**Table 3:** Worst performing keywords.

Language	Worst performing keywords	English translation
English	major depressive disorder symptoms	
	online depression test	
	depression screening	
	Take a depression test	
Russian	Имею ли я депрессию	Do I have depression
	дипрессия симптомы	depression^1^ symptoms
	Depressija	depression^2^
	депрессия тест бесплатно	depression test free
Chinese	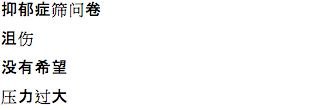	depression screening questionnaire
		depressed
		no hope
		excessive pressure
Spanish	Inútil y triste	useless and sad
	Prueba gratuita de depresión	free depression trial
	Deprezion	depression^1^
	Estado de ánimo triste	sad mood

^1^Deliberate misspellings in the original language.

^2^Transliteration.

**Table 4:** Best performing ads

Language	Best performing ads	English translations
English	Free depression test from the University of California, San Francisco	
Russian	Тест на депрессию. Узнайте есть ли у Вас депрессия. Бесплатный тест настроения из УКСФ	Test for depression. Find out if you have depression. Free mood test from UCSF.
Chinese	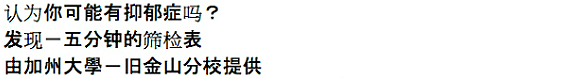	Do you think you might have depression? Find out – five minute survey provided by UCSF.
Spanish	Test de depresión gratis proporcionado por la Universidad de California, San Francisco.	Free depression test provided by the University of California, San Francisco.

**Table 5:** Worst performing ads.

Language	Worst performing ads	English translations
English	Can’t eat, sleep, focus? Find out if you are depressed with a free online test from UCSF.	
Russian	Проверка настроения. Узнайте есть ли у Вас депрессия. Бесплатный тест настроения из УКСФ.	Mood checker. Find out if you have depression. Free mood test from UCSF.
Chinese	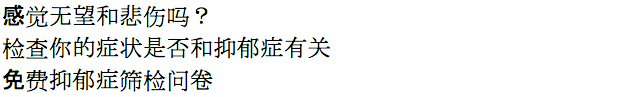	Feeling sad and hopeless? Check whether your symptoms are related to depression. Free depression questionnaire.
Spanish	Examine su ánimo. Complete gratis una evaluación de la Universidad de California, SF.	Examine your mood. Complete a free evaluation from the University of California, SF.

### Managed Period

**Figure 2:**
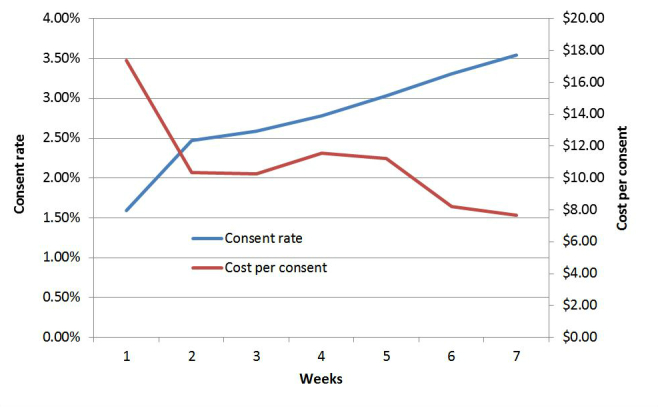
Weekly data on consent rate and per-consent cost during the managed period.

During the 7-week managed period, we monitored the campaigns closely, suspending underperforming ads and keywords and adding new keywords, with the goal of improving consent rate and reducing per-consent cost. Table 6 presents details on the first 2 weeks of the managed period and the last 2 weeks, to illustrate the changes. The number of impressions increased by 38.10%, clicks increased by 34.82%, conversions increased by 91.84% and the CR increased by 42.11%. The cost-per-click and cost-per-consent decreased by 16.13% and 23.31% respectively. The weekly changes in the two metrics most relevant to researchers planning to conduct AdWords campaigns—consent rate and per-consent costs—are illustrated in Figure 2.

**Table 6:** Changes in ad campaign performance during the managed period.

	First 2 weeks	Last 2 weeks	Change, %
Impressions, n	331,273	457,506	+38.10
Clicks (visitors), n	8,812	11,881	+34.82
Click-through rate, %	2.66	2.60	–2.26
Cost-per-click, $US	0.62	0.52	–16.13
Consented, n	184	353	+91.84
Consent rate, %	2.09	2.97	+42.11
Cost per consent, $US	18.23	13.98	–23.31

## Discussion

### Principal Findings

The purpose of this study was to describe worldwide Google AdWords campaigns recruiting participants to a health research website consisting of a depression screening study in four languages, to illustrate similarities and differences among the four languages and to demonstrate that active management of AdWords campaigns can substantially increase the recruitment rates while decreasing costs.

Our results offer a compelling illustration of the competitive nature of Google AdWords campaigns. Given the auction system that drives AdWords, popular markets such as the English-speaking market require greater expenditures to be competitive. The low rate of consent along with very high costs likely reflect the high competition for English-language depression-related keywords as well as perhaps the perception among English speakers that multiple options for information and monitoring are available to them. Not only are there more advertisers vying for the top spot in English-language ads, thereby increasing the price, but the market itself is saturated with ads and products that cater to English speakers, which results in users being both more selective and more accustomed to ignoring advertisements. These pressures likely resulted in English-language ads having the highest cost-per-click and by far the highest per-consent cost, which was about five times higher than any of the other three languages. Thus, a researcher wishing to conduct multilingual campaigns must consider not only the relative popularity (and therefore cost) of the keywords they plan to use to trigger the ads, but also the languages of the campaign itself, and to allocate funds appropriately to ensure equal recruitment.

Another difference that appeared to emerge in our data is the timing of individuals’ commitment, as evidenced chiefly by differences in the CTRs and CRs between Chinese and Russian ads. Whereas Russian speakers were quite willing to click on ads—their CTR was the highest of all languages—they appeared reluctant to commit to participate, exhibiting a very low consent rate. This suggests that, even though the initial interest in the site among Russian speakers was high, it applied only to the initial screening, and not to any further involvement. Chinese speakers, on the other hand, may be reluctant to visit an unknown site based on a simple ad, but once they visit, they are more likely to continue participating in the study. The results suggest that Chinese-speaking individuals looking for information on depression are quite selective as to which ad they respond to, but once they make a selection they are more likely to maintain their commitment. This suggests that both the sites and the ads may need to be structured differently depending on the language of the intended audience. For instance, a researcher might choose to design an especially attractive and thorough ad for Chinese speakers or might put more effort into the design of the recruitment message for Russian speakers.

There were notable similarities between the languages in regards to ads and keywords that were the most productive. For instance, ads briefly describing the purpose of the study (eg, depression screener) and using the word “free” resulted in highest CTRs and CRs. The ads that listed specific symptoms or ads that promoted self-improvement did not generate sufficient participant flow. This suggests that researchers wishing to simplify their ad campaigns should consider using simple, descriptive ads that are likely to be universally acceptable. Future studies might examine whether individuals with specific characteristics might be more likely to respond to a specific type of an ad. Keywords likewise revealed similarities, with all languages having “depression” or “depression test” as one of the most productive keywords. This observation was expected: individuals are more likely to click on an ad that conforms to the terms of their search. There were some notable departures from this pattern. First, keywords related to suicide generated considerable participant flow among Chinese-speakers (and among Russian-speakers as well). The fact that Chinese speakers who searched for suicide-related terms clicked on an ad for a depression-related site might suggest that these individuals are particularly troubled by suicidal symptoms [16]. Alternatively, this might suggest the paucity of resources available online for Chinese and Russian speakers (for instance, searching for the word “depression” in English produces 62 million results, compared to 15 million results for the Chinese term for depression and less than 3 million for Russian). The same may be true for Spanish speakers (only 1.5 million results for the Spanish word for depression), for whom “la ansiedad” (anxiety) was one of the top keywords that led individuals to click on an ad for a depression screening study.

Importantly, we found that that by actively managing AdWords campaigns, it is possible to both increase the rate of recruitment and decrease costs. Our strategy targeted both ads and keywords. We removed under-performing keywords in an attempt to weed out individuals who may be less likely to participate (based on data from previous visitors using the same keywords to enter the study). We also removed under-performing ads to ensure that individuals would see ads that were more likely to bring them into the study. These efforts resulted in higher traffic and lower per-person costs. In the era of shrinking research budgets, being able to significantly increase the consent rate while decreasing per-consent cost should be a relief for researchers attempting to do large-scale recruitment with Google AdWords. Although Google AdWords has built-in tools to improve ad performance, researchers who wish to maximize the efficiency of their ad campaigns are advised to actively manage and optimize their ad campaigns. No person-hours were spent during the 6-month “Unmanaged” campaign. During the 7-week managed period, a research assistant spent approximately 1.5-2 hours per week examining the campaigns, pausing keywords and ads, and adding keywords. Slightly more time was spent during the beginning weeks of the managed period to make adjustments to the campaigns, and time commitments lessened somewhat during the final weeks. The personnel cost, however, was offset by the greater efficiency and effectiveness of the recruitment campaigns, and we believe that this investment in person-hours is well justified.

### Limitations

This study has several limitations. The study describes two distinct recruitment periods that took place 1 year apart, with the managed period being conducted in the spring, while the unmanaged period was conducted over winter and the beginning of spring. It is possible that seasonal differences could have affected the flow and the type of participants searching depression-related terms. The numbers from these periods are not directly comparable given the rapid changes in Internet and its users and in Internet advertisement. To illustrate changes in ad performance, we selected a short management period (7 weeks) to minimize the historical influence of changes in Internet and Internet advertisement. We report changes in ad performance in a “pre-post” design; conducting two campaigns simultaneously, one managed and one unmanaged, would have been a more powerful illustration. However, such a design may introduce considerable undesirable confounds as ads in such two campaigns would be competing against each other. For Chinese and Russian speakers, Google is not the dominant search engine (Baidu and Yandex have larger market shares in China and Russia, respectively), thus our data may not be fully representative of Chinese- and Russian-speaking Internet users. Given the evolving nature of Internet users, the specifics of our results may not reflect future Internet users; however, our conclusions regarding the need to monitor and carefully manage ad campaigns to optimize their performance will most likely remain applicable well into the future.

### Conclusions

The Internet made it possible to conduct research in the global community, on populations that would otherwise not be represented in research samples, and to do so quickly, efficiently, and inexpensively. Researchers who wish to take advantage of such tremendous research opportunities can make use of sophisticated tools specifically designed to reach individuals on the Internet and deliver targeted recruitment messages. The enormous diversity of global populations calls for a similar diversity of outreach, both to make research opportunities appealing to as many people as possible, as well as to increase the efficiency of such endeavors. This study demonstrates the potential of a tool such as Google AdWords for recruitment of research participants, illustrates the differences in effectiveness of recruitment campaigns between four different languages, and emphasizes the value of actively managing recruitment. Understanding both differences and similarities of outreach approaches will help advance research practices and, consequently, the research itself.

## Abbreviations

CR: conversion rate
CTR: click-through rate
DSM-IV: Diagnostic and Statistical Manual of Mental Disorders, 4th edition
MDE: major depressive episode
PRIME-MD: Primary Care Evaluation of Mental Disorders
